# Cardiac dysfunction during immune checkpoint inhibitor therapy: association with extracardiac immune-related adverse events

**DOI:** 10.1186/s40959-026-00529-4

**Published:** 2026-06-16

**Authors:** Raluca I. Mincu, Lena Lampe, Lars Michel, Amir A. Mahabadi, Adelina V. Mark, Lisa Zimmer, Elisabeth Livingstone, Dirk Schadendorf, Alpaslan Tasdogan, Tienush Rassaf, Matthias Totzeck

**Affiliations:** 1 Department of Cardiology and Vascular Medicine, West German Heart and Vascular Center Essen, University Hospital Essen, Hufelandstrasse 55, Essen, 45147 Germany; 2 Department of Dermatology, West German Skin Tumor Center Essen, University Hospital Essen, Essen, Germany

**Keywords:** Immunotherapy, Heart failure, Echocardiography, Guidelines

## Abstract

**Background and aims:**

With the increasing approval of immune checkpoint inhibitors (ICI) for cancer treatment, there is a rising incidence of potentially life-threatening cancer-therapy related cardiac dysfunction (CTRCD). Current 2022 European Society of Cardiology (ESC) guidelines on cardio-oncology recommend a single baseline transthoracic echocardiogram (TTE) for high-risk ICI-treated patients, potentially underestimating the incidence of CTRCD due to lack of follow-up assessments. This study aims to characterize the incidence of CTRCD in cancer patients undergoing ICI therapy using an intensified TTE surveillance approach and to investigate whether extracardiac immune-related adverse events (eirAEs) are associated with CTRCD.

**Methods:**

We analysed patients scheduled for ICI therapy from the Essen Cardio-Oncology Registry (EcoR). Data were collected during cardio-oncology consultations at baseline, 6 weeks, 6 months, and 12 months. Patients with eirAEs were evaluated compared to patients without eirAEs.

**Results:**

Among 2,540 cancer patients registered in EcoR until march 2023, 266 (61 ± 14 years, 40.6% female, 86.5% melanoma, 62% metastatic disease) were scheduled for ICI therapy. CTRCD was observed in 35.7% of patients, with 32.7% having asymptomatic mild CTRCD, 0.37% moderate CTRCD, and 2.63% developing heart failure with preserved ejection fraction (HFpEF). Patients with eirAEs had a twofold higher risk of CTRCD (relative risk [RR] 2.05, 95% CI: 1.49–2.83, *p* < 0.001) and a 66% increased risk of cardiovascular toxicity (RR 1.66, 95% CI 1.30–2.13, *p* < 0.001).

**Conclusions:**

ICI therapy is associated with a high prevalence of CTRCD, underscoring the importance of serial echocardiography evaluation, particularly for patients with eirAEs.

Enhanced cardio-oncology surveillance is crucial for improving patient outcomes and survival.

**Supplementary Information:**

The online version contains supplementary material available at 10.1186/s40959-026-00529-4.

## Introduction

ICI therapy has revolutionized cancer treatment, significantly improving survival [1–4]. With more than one in three cancer patients being eligible for ICI therapy, and the continuous development of new ICIs with potential cardiovascular toxicity, the urgent need for a comprehensive, proactive, and specialized cardio-oncology surveillance strategy is evident [5]. 

ICI therapy enhances antitumor immunity by blocking intrinsic immune downregulators such as cytotoxic T-lymphocyte antigen 4 (CTLA-4) and programmed cell death protein 1 (PD-1) or its ligand, programmed death-ligand 1 (PD-L1) [6]. The activation of immunity during ICI therapy may trigger the development of both cardiac and eirAEs, leading to significant morbidity and mortality [7]. While ICI-induced myocarditis remains the most studied cardiovascular toxicity, emerging clinical and preclinical data highlight the importance of other cardiotoxicities, including CTRCD [8]. EirAEs, affecting the gastrointestinal tract, endocrine glands, skin, and liver, occur in more than 30% of patients treated with ICIs and can involve virtually all organs and systems [6, 9–11]. However, whether eirAEs are associated with the development of CTRCD remains unknown, representing a critical gap in the understanding of ICI-associated cardiovascular toxicity.

The true incidence of heart failure in ICI-treated patients remains poorly defined, as cardiovascular toxicities are often reported as composite endpoints [12, 13]. While some studies estimate a 1.6% to 2% incidence of heart failure under ICI therapy [14, 15], a recent meta-analysis suggests rates of 0.80% with PD-1/PD-L1 inhibitors and 1% with the CTLA-4 inhibitor ipilimumab [16]. However, asymptomatic CTRCD may be significantly more common, with reported rates ranging from 8.5% to 31.3% in ICI-treated patients across two studies [15, 17]. These findings suggest that CTRCD is vastly underdiagnosed and may be overlooked due to the lack of structured follow-up assessments.

Furthermore, the underlying pathophysiology of CTRCD under ICI therapy remains poorly understood [18]. Our research group previously demonstrated that anti-PD-1 therapy in a murine melanoma model led to early CD4 + and CD8 + T cells myocardial infiltration, dysregulation of proteasome and lipid metabolism, and myocardial dysfunction, alongside PD-L1 upregulation on cardiac endothelial cells. In line with these findings, patients with metastatic melanoma receiving anti-PD-1 therapy exhibited impaired left ventricular function during dobutamine stress testing [19]. These insights indicate a direct mechanistic link between ICI therapy and myocardial dysfunction, further underscoring the urgent need for systematic cardiovascular monitoring.

Despite of the central role of transthoracic echocardiography (TTE), especially of the left ventricular ejection fraction (LVEF) and global longitudinal strain (GLS) in the diagnosis of CTRCD, the actual surveillance protocols for patients under ICI therapy indicate the evaluation trough TTE only at baseline in patients at high risk to develop cardiovascular toxicity, without any follow-up examinations. Patients at high risk for ICI-associated cardiovascular toxicity include those receiving dual ICI therapy (e.g., the CTLA-4 inhibitor ipilimumab and the PD-1 inhibitor nivolumab), those undergoing combination therapy with ICI and other cardiotoxic treatment, as well as patients who have experienced eirAEs, prior cancer therapy-associated cardiac dysfunction, or pre-existing cardiovascular disease. TTE in patients at low risk to develop cardiotoxicity should not be performed [20]. Without systematic echocardiographic follow-up, the early detection of CTRCD is impossible, potentially compromising patient outcomes.

We aim to characterize the incidence of CTRCD in cancer patients undergoing ICI therapy when echocardiographic evaluations are performed more frequently than currently recommended and to determine whether eirAEs are associated with the development of CTRCD, providing critical insights into the interplay between extracardiac and cardiac immune-related toxicities.

By addressing these urgent research gaps, we seek to develop new cardio-oncology surveillance protocols, advocating for the routine integration of serial echocardiographic monitoring in ICI-treated patients—especially those experiencing eirAEs. A failure to act on this knowledge may result in missed opportunities for early intervention, significantly impacting cancer survival and long-term cardiovascular outcomes.

## Methods

### Study population

We selected all consecutive cancer patients scheduled to receive ICI therapy from our local EcoR database. The patients have been referred to our cardio-oncology outpatient unit between July 2018 until March 2023 for evaluation before initiation of the ICI therapy and were under cardio-oncology surveillance during ICI therapy. The registry was initiated to include all cancer patients evaluated in the cardio-oncology unit at our tertiary University Hospital Center, in order to characterize cardiovascular toxicity. The study was approved by the local ethics committee (No. 19-8632-BO). The patients were evaluated according to the local protocol at baseline (before ICI therapy initiation), at 6 weeks, 6 months and 12 months after initiation of ICI therapy. Individual informed consent was not required for this retrospective analysis of anonymized data, in accordance with local ethical guidelines and with approval from the institutional ethics committee. Data regarding cancer types, specific oncology treatments, previous oncologic surgery, radiation therapy was also extracted.

Transthoracic echocardiography was performed using a commercially available ultrasound system (EPIQ 7 C/CVx, Philips Healthcare, Andover, MA, USA), equipped with advanced cardiac imaging capabilities, including Dynamic Heart Model for the assessment of 3D LVEF.

Parameter of systolic and diastolic function measured according to the actual guidelines of the American Society of Echocardiography and European Association of Cardiovascular Imaging [21]. GLS, global radial strain (GRS) and global circumferential strain (GCS) were calculated using speckle tracking method using QLAB software (version 10, Philips Healthcare, Andover, MA, USA) [22]. 

In order to study the association between eirAEs and CTRCD, we divided the study population into two groups: group 1 patients with eirAEs and group 2 with patients without eirAEs during the treatment. The patients in group 1 and group 2 were not matched on any characteristics. 

### Primary endpoint

We aimed to assess the incidence of CTRCD in our study population and evaluate whether patients with eirAEs have a higher incidence of CTRCD compared to those without eirAEs, utilizing a local echocardiography follow-up protocol. CTRCD was defined in accordance with established 2022 ESC guidelines on cardio-oncology (see Supplementary Table 1) [20].

Extracardiac immune-related adverse events (eirAEs) were defined and graded by the treating oncologists according to the Common Terminology Criteria for Adverse Events (CTCAE), version 5.0. 

### Secondary endpoint

As secondary endpoint we assessed the incidence of ICI myocarditis, arrhythmia and QTc prolongation and vascular toxicity. A composite secondary endpoint “cardiovascular toxicity” was defined based on 2022 ESC guidelines on cardio-oncology-defined toxicities, including CTRCD, myocarditis, arrhythmias, and vascular toxicity. No additional cardiac dysfunction classification was applied in patients diagnosed with myocarditis. The diagnosis of all these cardiovascular toxicities was performed according to the 2022 ESC guidelines on cardio-oncology guidelines definitions (Supplementary Table 1) [20]. 

### Statistical analysis

Continuous variables are reported as mean ± standard deviation (SD) or median and interquartile range (IQR) and categorical variables are reported as number of patients and percentages. Comparisons between group 1 and group 2 were done with Student’s T test or Wilcoxon-Mann-Whitney-Test for continuous variables, while for categorical variables Fischer’s exact test or chi-square was used. The proportion of patients with adverse events in the two groups is expressed as the relative risk (RR) and 95% confidence interval (CI). Values of GLS, GRS, GCS, 2D LVEF and 3D LVEF at different timepoints within the two groups were compared with paired samples T test. Survival curves were computed through Kaplan-Meier analysis using the log-rank test for 1-year survival.

All analyses were performed using IBM SPSS Statistics Version 27. Differences with p-values ≤ 0.05 (2-sided) were considered statistically significant.

## Results

### Study population

Out of 2,540 cancer patients in our EcoR database, 520 received ICI therapy. Of these, 254 were excluded due to the absence of a cardio-oncology evaluation before starting treatment.

**Fig. 1:**
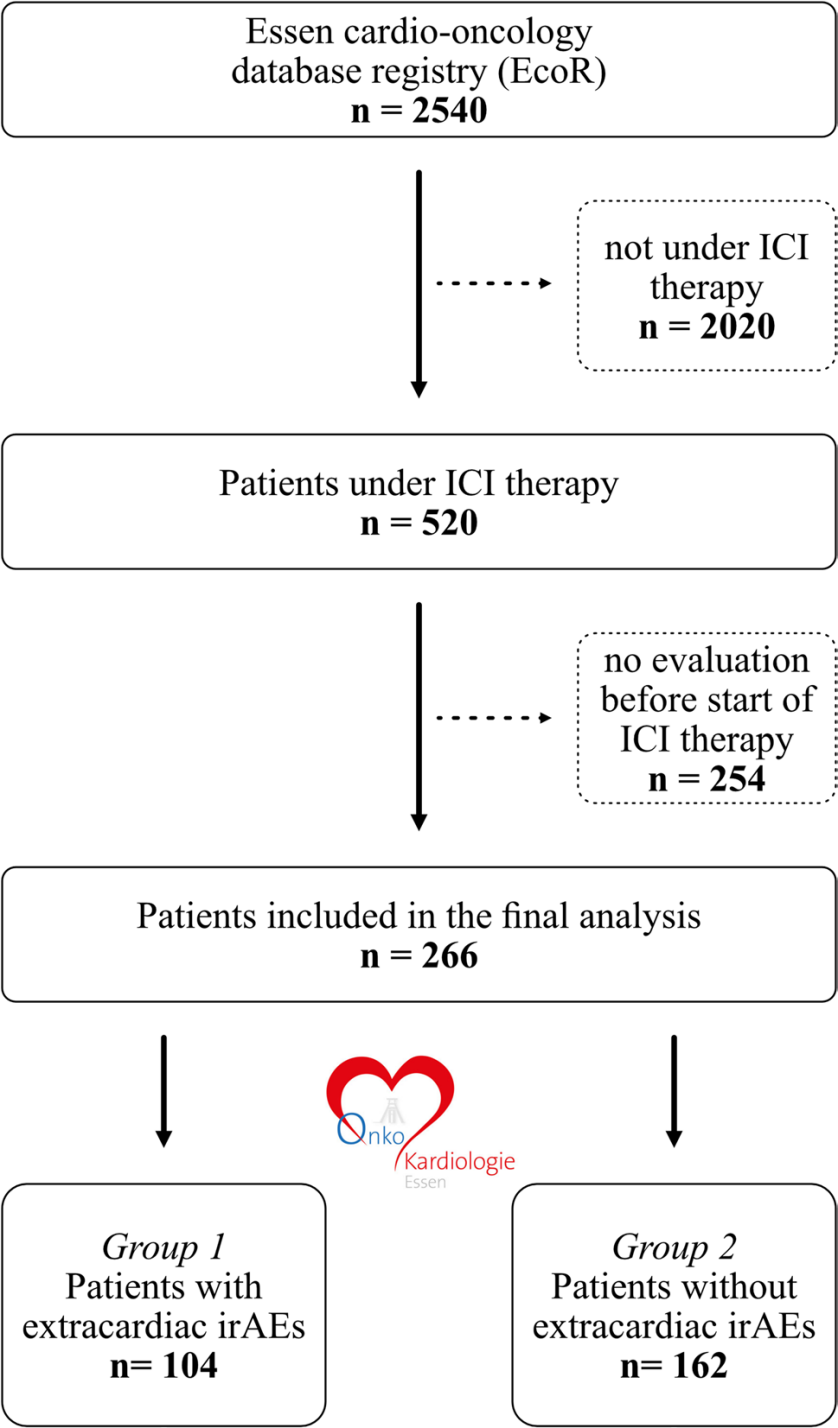
Flowchart for patient selection. Out of 2,540 cancer patients in our EcoR database, 520 received ICI therapy. Of these, 254 were excluded due to the absence of a cardio-oncology evaluation before starting treatment. The final study cohort included 266 cancer patients scheduled for ICI therapy. These patients were divided into two groups: 104 who developed eirAEs during ICI therapy and 162 who did not. eirAE = extracardiac immune-related adverse events; ICI = immune checkpoint inhibitors

The final study cohort included 266 cancer patients scheduled for ICI therapy. For the subgroup analysis we divided the patients into two groups: 104 who developed eirAEs during ICI therapy and 162 who did not (Fig. 1).

### Baseline characteristics of the study population

The baseline characteristics of the entire study population are depicted in Table 1. Patients were 61 ± 14 years old, 40.6% were female. The most frequent cardiovascular risk factor was arterial hypertension. 25,6% of the entire study population had a history of cardiovascular disease. Patients in the group 1 (with eirAEs) and group 2 (without eirAEs) were comparable in terms of age, gender distribution, and cardiovascular risk factors. Additionally, there were no significant differences in the medical history of cardiovascular, pulmonary, or renal diseases between the groups (Table 1).

**Table 1:** Baseline characteristics of the entire study population and of the two study groups

	*n* = 266	Group 1 Patients with eirAEs *n* = 104	Group 2 Patients without eirAEs *n* = 162		*p*-value group 1 vs. group 2	
Baseline characteristics						
Demographics						
Age (years)	61 ± 14	61 ± 13	62 ± 14		0.412	
Female (%)	108 (40.6)	45 (43.2)	63 (38.8)		0.478	
Cardiovascular risk factors or medical history						
Obesity n (%)	71 (26.6)	21 (21.4)	50 (32.4)		0.062	
Dyslipidemia n (%)	38 (14.5)	14 (13.5)	24 (14.8)		0.758	
Arterial hypertension n (%)	154 (57.8)	55 (52.9)	99 (61.9)		0.148	
Smoking n (%)	39 (14.7)	15 (14.4)	24 (14.9)		0.914	
Diabetes mellitus n (%)	43 (16.2)	13 (12.5)	30 (18.5)		0.193	
Any history of cardio-vascular disease n (%)	69 (25.9)	24 (23.1)	45 (27.8)		0.393	
Chronic coronary syndrome n (%)	35 (13.2)	10 (9.6)	25 (15.4)		0.171	
History of myocardial infarction n (%)	13 (4.9)	2 (2.9)	10 (6.2)		0.231	
History of percutaneous coronary intervention n (%)	25 (9.4)	8 (7.7)	17 (10.5)		0.445	
History of coronary artery by-pass grafting n (%)	9 (3.4)	1 (1)	8 (5)		0.077	
Heart failure n (%)	19 (7.1)	6 (5.8)	13 (8)		0.486	
Chronic obstructive pulmonary disease n (%)	10 (3.8)	3 (2.9)	7 (4.3)		0.548	
Peripheral artery disease n (%)	4 (1.5)	2 (1.9)	2 (1.2)		0.653	
Moderate valvular heart disease n (%)	15 (5.7)	7 (6.8)	8 (5)		0.532	
Atrial fibrilation n (%)	26 (9.8)	7 (6.7)	19 (11.8)		0.175	
History of stroke n (%)	14 (5.3)	7 (6.7)	7 (4.3)		0.390	
Chronic kidney disease n (%)	12 (4.5)	9 (2.9)	3 (5.6)		0.313	
Echocardiographic parameters baseline						
LVEF < 40% n (%)	5 (2)	2 (2)	3 (1.9)		0.977	
LVEF 40–50% n (%)	11 (4.3)	2 (2)	9 (5.9)		0.139	
LVEF > 50% n (%)	244 (91.7)	99 (95.2)	145 (89.5)		0.100	
High risk n (%)	123 (46.2)	51 (49)	72 (44.4)		0.463	
Troponin I (ng/l)	4 (3–5)	3 (3–9.25)	5 (3–9)		0.108	
NT-proBNP (pg/ml)	203 (167–316)	163 ( 67.5–659)	306.5 (109.75–909.75)		0.055	
Tumor entity and therapy regime						
Tumor entity						
Malignant melanoma n (%)	230 (86.5)	100 (96.1)		130 (80.2)		0.001
Merkel cell carcinoma n (%)	11 (6.8)	2 (1.9)		9 (5.6)		0.147
Squamous cell skin cancer n (%)	18 (6.8)	1 (1)		17 (10.5)		0.003
Hepatocellular carcinoma n (%)	1 (0.4)	0 (0.0)		1 (0.6)		0.421
Non-small cell lung cancer n (%)	3 (1.1)	1 (1.0)		2 (1.2)		0.837
Clear cell renal cancer n (%)	1 (0.4)	0 (0.0)		1 (0.6)		0.422
Urothelial carcinoma n (%)	2 (0.8)	0 (0.0)		2 (1.2)		0.255
Local or advanced disease						
Metastasis n (%)	165 (62.0)	75 (72.1)		90 (55.6)		0.007
Recurrent disease n (%)	173 (65.0)	79 (76.0)		94 (58.0)		0.003
Palliative care n (%)	35 (13.2)	14 (13.5)		21 (13.0)		0.907
≥ 2 tumor entities (in the last 5 years) n (%)	24 (9.0)	5 (4.8)		19 (11.7)		0.055
Time since diagnosis (months)	40.2 ± 40.6	38,9 ± 35,8		41.7 ± 40.2		0.628
Tumor progress n (%)	47 (17.7)	21 (20.2)		26 (16.0)		0.387
Therapy						
Previous surgery n (%)	260 (97.7)	104 (100.0)		156 (96.3)		0.047
Surgery after ICI therapy start n (%)	33 (12.5)	18 (17.5)		15 (9.3)		0.048
Previous radiation therapy n (%)	70 (26.4)	32 (30.8)		38 (23.6)		0.196
Pervious chest irradiation n (%)	14 (5.3)	4 (3.8)		10 (6.2)		0.401
Previous chemotherapy n (%)	9 (3.4)	3 (2.9)		6 (3.7)		0.718
Anthracycline n (%)	1 (0.4)	1 (1.0)		0 (0.0)		0.211
Platinum based chemotherapy n (%)	7 (2.6)	2 (1.9)		5 (3.1)		0.563
Alkylating agents n (%)	4 (1.5)	2 (1.9)		2 (1.2)		0.653
BRAF inhibitors n (%)	20 (7.5)	7 (6.7)		13 (8.0)		0.696
MEK inhibitors n (%)	19 (7.1)	7 (6.7)		12 (7.4)		0.834
Tyrosine kinase inhibitors n (%)	1 (0.4)	1 (1.0)		0 (0)		0.211
Nivolumab n (%)	200 (75.5)	92 (88.5)		108 (67.1)		< 0.001
Pembrolizumab n (%)	59 (22.5)	22 (21.4)		37 (23.3)		0.718
Cemiplimab n (%)	19 (7.3)	1 (1)		18 (11.3)		0.002
CTLA4 Inhibitor (Ipilimumab) + Nivolumab n (%)	67 (25.5)	35 (34.0)		31 (20.0)		0.011

Shown are the baseline characteristics of the entire group and of the group 1 of patients who experienced extracardiac immune related adverse events (eirAEs) in comparison with group 2 of patients who did not experience eirAEs. Additionally, the table shows the distribution of tumor entities, their characteristics and the treatment regimen in the entire group and in group 1 in comparison with group 2. Values are expressed in number of patients (percent of total patient population) n (%). Data represent mean ± SD or number of patients (percent of total patient population) n (%)

*eirAEs*  Extracardiac immune related adverse events, *LVEF*  Left ventricular ejection fraction, *BRAF*  Rapidly accelerated fibrosarcoma Isoform B, *CTLA-4*  Cytotoxic T-lymphocyte antigen 4, *eirAE*  Immune related adverse events, *ICI*  Immune checkpoint inhibitor, *PD-1*  Programmed cell death 1, *PD-L1*  Programmed cell death ligand 1, *MEK*  Mitogen-activated protein kinase

*p*-values are two-sided

Of the 266 patients, 230 (86.5%) had malignant melanoma, followed by cases of Merkel cell carcinoma, squamous cell skin cancer, and hepatocellular carcinoma. Patients with eirAEs had a significantly higher incidence of metastatic disease (72.1% vs. 55.6%, p = 0.007). The distribution of radiotherapy and chemotherapy was similar between the groups. However, treatment with nivolumab or a combination of nivolumab and ipilimumab was significantly more common in the eirAEs group (88.5% vs. 67.1%, p < 0.001; 34% vs. 20%, p = 0.011, respectively) (Table 1).

The distribution of eirAEs is detailed in the appendix (supplementary Table 2). The most common eirAE in Group 1 was hepatitis, followed by colitis, thyroiditis, hypophysitis, and pneumonitis. Additionally, 20 patients (38.4%) experienced more than one eirAE (supplementary Table 3).

### Dynamic of LVEF and GLS during ICI therapy

**Fig. 2:**
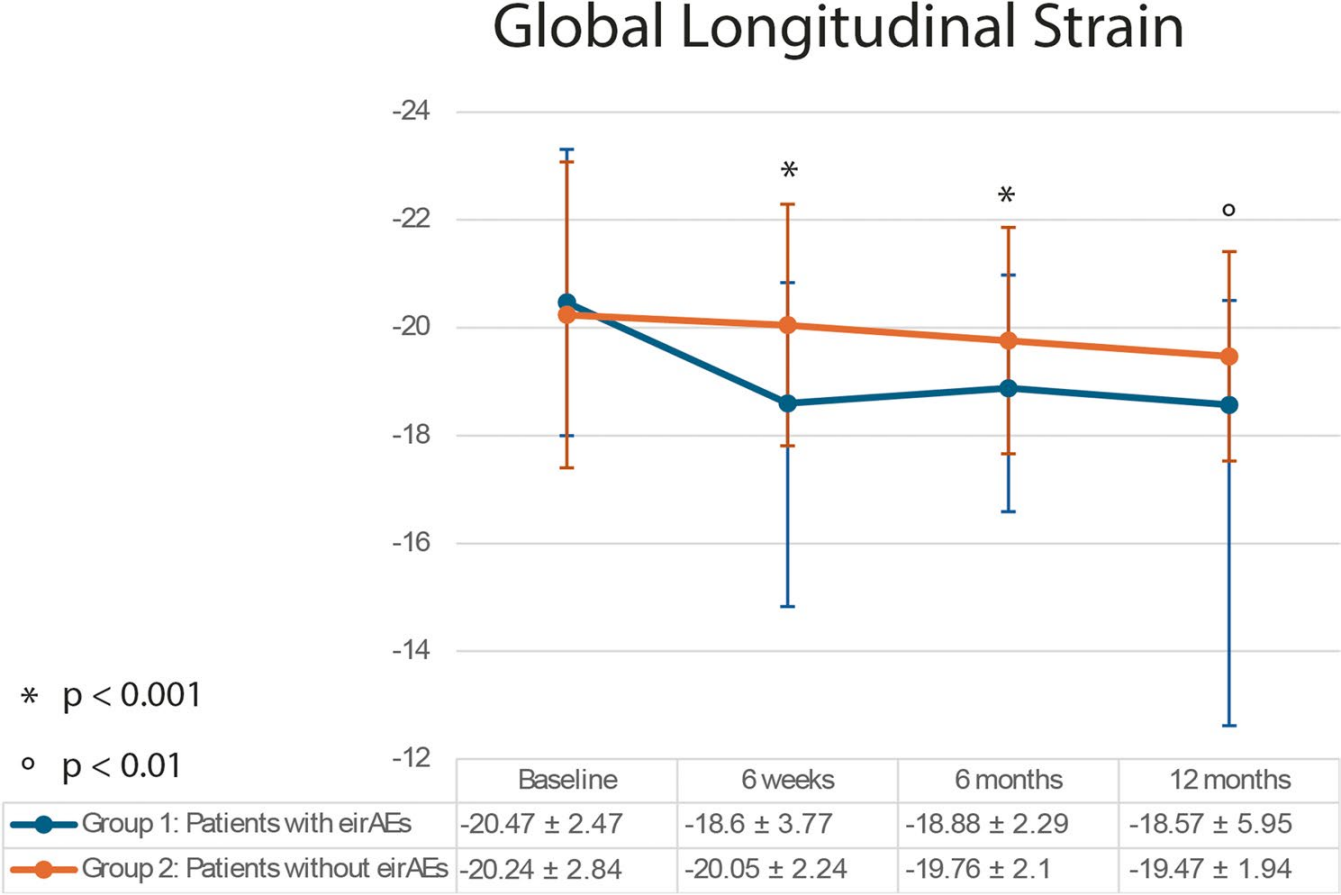
Temporal trends in global longitudinal strain (GLS) measurements in the two study groups. The figure shows temporal trends in mean values and standard deviations of global longitudinal strain (GLS) at baseline, 6 weeks, 6 months and 12 months follow-up echocardiography in the two study groups

There was no difference regarding both bidimensional or tridimensional LVEF at the three different timepoints between the two groups. Patients with eirAEs showed a significant reduction of GLS at 6 weeks follow-up (-20.47 ± 2.47 vs. -18.60 ± 3.77, *p* < 0.001), 6 months follow-up (-20.47 ± 2.47 vs. -18.88 ± 2.29, *p* < 0.001), and 12 months follow-up -20.47 ± 2.47 vs. -18.57 ± 5.95, *p* < 0.001). This phenomenon was not observed in the group of patients without eirAEs (Fig. 2). GRS and GCS remained unchanged during the therapy (supplementary Table 3).

### CTRCD under ICI therapy

**Fig. 3:**
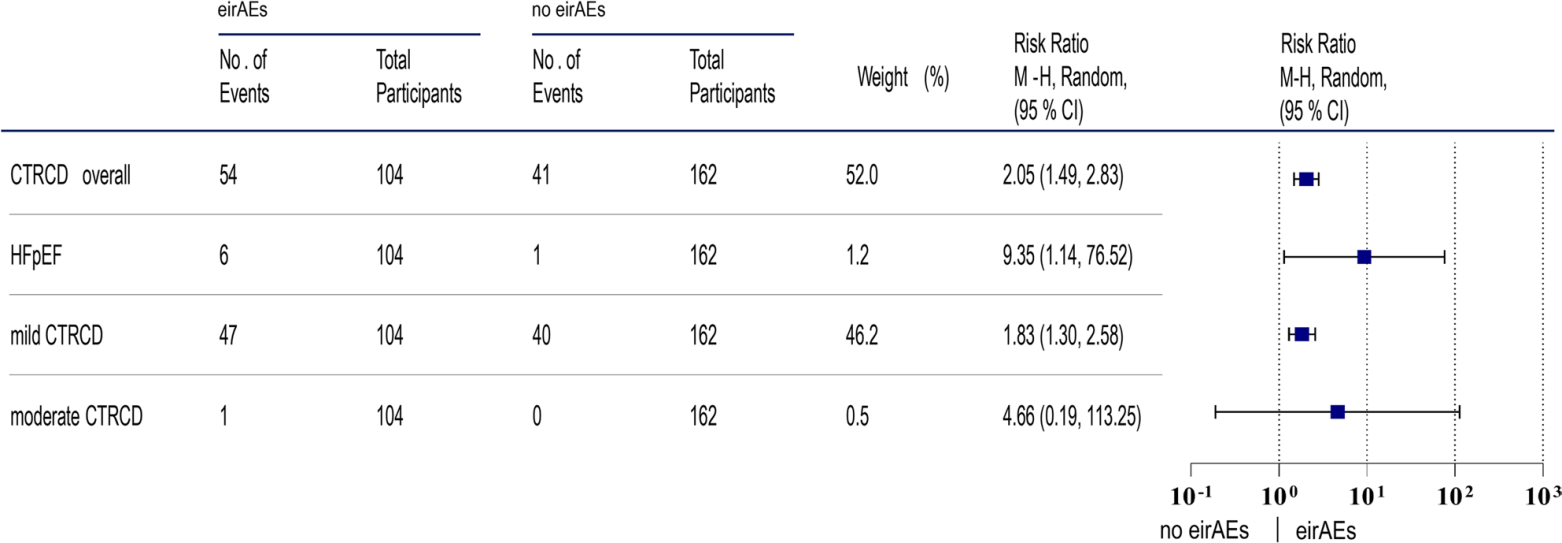
Relative risk of CTRCD in the group of cancer patients under ICI therapy with eirAEs compared to cancer patients without eirAEs. Forest plot showing the RR of CTRCD in patients with eirAEs compared to patients without eirAEs. Patients with eirAEs had significantly higher risks of overall CTRCD (RR 2.05), mild CTRCD (RR 1.83), and HFpEF (RR 9.35). CTRCD = cancer therapy related cardiac dysfunction; ICI = immune checkpoint inhibitors; eirAEs = extracardiac immune-related adverse events; HFpEF = heart failure with preserved ejection fraction; RR = relative risk

The incidence of CTRCD was notably high, with 35.7% of patients developing CTRCD. Among them, 32.7% had asymptomatic mild CTRCD, 0.37% had asymptomatic moderate CTRCD, and 2.63% developed HFpEF (Table 2). Patients with eirAEs had a twofold higher risk of developing CTRCD overall (relative risk [RR] 2.05, 95% CI: 1.49–2.83, *p* < 0.001), an overall 83% increased risk of asymptomatic mild CTRCD (RR 1.83, 95% CI: 1.30–2.58, *p* < 0.001) and a more than ninefold risk of HFpEF (RR 9.35, 95% CI: 1.14–76.52, *p* = 0.040) (Table 2; Fig. 3). The diagnosis of CTRCD was established according to the 2022 ESC guidelines on cardio-oncology definitions. Overall, 33.6% of patients with CTRCD (32 patients) were diagnosed based on a ≥ 15% relative reduction in GLS, comprising 24 patients in the eirAEs group and 8 patients in the non-eirAEs group. In 57.8% of cases (55 patients), diagnosis was based on changes in NT-proBNP levels, including 20 patients with eirAEs and 35 without. In the remaining 8.4% (8 patients), both GLS reduction and NT-proBNP elevation contributed to the diagnosis—all of whom were in the eirAEs group.

**Table 2:** Immune checkpoint inhibitor related cardiovascular toxicity in the entire study population and in the two groups

Outcome		Overall *n* = 266	Group 1 eirAEs (*n* = 104)	Group 2 no eirAEs (*n* = 162)	Risk ratio [95% CI]	*p*-value group 1 vs. group 2
Overall cardiovascular toxicity		126 (47.3)	65 (62.5)	61 (36.74)	1.66 [1.30, 2.13]	< 0.001
More than one cardiovascular toxicity		37 (13.9)	25 (24.03)	12 (7.22)	3.25 [1.71, 6.17]	< 0.001
CTRCD	overall	95 (35.71)	54 (51.92)	41 (25.30)	2.05 [1.49, 2.83]	< 0.001
	asymptomatic mild	87 (32.70)	47 (50.96)	40 (24.69)	1.83 [1.30, 2.58]	< 0.001
	asymptomatic moderate	1 (0.37)	1 (0.96)	0 (0)	4.66 [0.19, 113.25]	0.213
	asymptomatic severe	0 (0)	0 (0)	0 (0)	n. a.	n. a.
	HFpEF	7 (2.63)	6 (5.76)	1 (0.61)	9.35 [1.14, 76.52]	0.040
Myocarditis	non-fluminant	11 (4.13)	11 (10.57)	0 (0.0)	35.70 [2.13, 599.48]	< 0.001
Arrythmia or QTc prolongation	overall	47 (17.66)	24 (23.07)	23 (13.85)	1.63 [0.97, 2.72]	0.07
	sinus tachycardia	13 (4.88)	8 (7.69)	5 (3.08)	2.49 [0.84, 7.41]	0.080
	sinus bradycardia	14 (5.26)	7 (6.73)	7 (4.32)	1.56 [0.56, 4.31]	0.364
	atrial fibrillation	7 (2.63)	2 (1.92)	5 (3.08)	0.62 [0.12, 3.15]	0.583
	ventricular premature beats	5 (1.87)	3 (2.88)	2 (1.20)	2.34 [0.40, 13.75]	0.319
	QTc prolongation	8 (3.00)	4 (3.84)	4 (2.40)	1.56 [0.40, 6.09]	0.498
Vascular Toxicity	overall	18 (6.76)	8 (7.69)	10 (6.02)	1.25 [0.51, 3.05]	0.615
	myocardial infarction	7 (2.63)	2 (1.92)	5 (3.01)	0.62 [0.12, 3.15]	0.571
	stroke	1 (0.37)	0 (0.0)	1 (0.6)	0.52 [0.02, 12.58]	0.424
	deep vein thrombosis	7 (2.63)	4 (3.84)	3 (1.80)	2.08 [0.47, 9.09]	0.315
	pulmonary embolism	4 (1.5)	3 (2.88)	1 (0.60)	4.67 [0.49, 44.33]	0.135

The table shows the cardiovascular toxicity in the study population and in the two groups: group 1: patients with irAEs, group 2: patients without irAEs.  Values are expressed in number of patients (percent of total patient population) n (%)

*CTRCD* Cancer therapy related cardiac dysfunction, *eirAEs*  Extracardiac immune related adverse events

Overall, patients with eirAEs experienced 66% more cardiovascular toxicity compared to those without eirAEs (RR 1.66, 95% CI 1.30–2.13, *p* < 0.001). Additionally, the RR of more than one cardiovascular toxicity was 3.25 times higher in the eirAE group (Table 2).While CTRCD and myocarditis were significantly more frequent in patients with eirAEs, the incidence of vascular toxicity and arrhythmias remained similar between the two groups (Table 2). There were no differences regarding one year survival between the two groups (86.4% vs. 88.3%, *p* = 0.671). Survival data for the 254 excluded patients are available. The survival curve of ICI therapy–naive patients differed significantly from that of non–therapy-naive patients. After one year, 86.5% of therapy-naive patients were still alive, wheareas only 77.8% of non–therapy-naive patients were alive (*p* = 0.002). (Supplementary Fig. 1).

## Discussion

We analyzed a real-world cohort of 266 cancer patients scheduled to receive ICI therapy from our EcoR database, assessing them at four key time points: before treatment initiation, and at 6 weeks, 6 months, and 12 months post-therapy. Patients were categorized into two groups: those who developed eirAEs and those who did not.

The study highlights the high incidence of CTRCD in patients undergoing ICI therapy. A total of 35.7% of patients developed CTRCD, with 32.7% experiencing asymptomatic mild CTRCD (42% diagnosed via a 15% relative reduction in GLS), 0.37% developing asymptomatic moderate CTRCD, and 2.63% presenting with HFpEF. In this cardio-oncology cohort of ICI-treated patients, we found that guideline-defined CTRCD occurred in one-third of individuals, with most cases representing mild, asymptomatic dysfunction. Importantly, patients who experienced extracardiac immune-related adverse events exhibited a markedly higher incidence of CTRCD and a substantially increased risk of HFpEF. These findings support the concept that immune-mediated cardiovascular injury may develop as part of a broader systemic inflammatory response triggered by immune checkpoint modulation.

These results reinforce the necessity of serial TTE evaluations as a standard diagnostic approach for patients under ICI therapy. Patients with eirAEs had a twofold higher risk of developing CTRCD overall, an overall 83% increased risk of asymptomatic mild CTRCD and a more than ninefold risk of HFpEF. Furthermore, patients who developed eirAEs exhibited significantly higher cardiovascular toxicity rates, with a 66% increased overall risk and a more than three-fold higher relative risk to develop more than one cardiovascular toxicity compared to those without eirAEs. Importantly, while CTRCD and myocarditis were significantly more common in patients with eirAEs, the rates of vascular toxicity and arrhythmia remained similar between the two groups. Patients with eirAEs had a higher incidence of metastatic disease and were more frequently treated with nivolumab-ipilimumab combination therapy. The most frequently observed eirAEs were hepatitis, colitis, and thyroiditis.

These findings align with and expand upon prior research on ICI-related cardiovascular toxicity. The underlying mechanisms of ICI-induced cardiovascular toxicity remain incompletely understood, but chronic inflammation and immune dysregulation appear to play central roles. Preclinical models suggest that anti-PD-1 therapy promotes myocardial infiltration by CD4 + and CD8 + T cells, disrupting proteasome function and lipid metabolism, ultimately leading to cardiac dysfunction [19]. Additionally, preexisting cardiac dysfunction and inflammatory triggers from other affected tissues, like during eirAE, may amplify the risk of immune-mediated myocardial damage [23]. 

The true incidence of CTRCD under ICI therapy remains incompletely characterized, with reports varying widely and with heart failure reported as composite endpoint. A Danish cohort study demonstrated an increased risk of cardiac events in melanoma and lung cancer patients treated with ICIs, with 1-year cardiac event risks of 6.6% and 7.5%, respectively [12]. In a retrospective study, heart failure was more common as a late ICI-associated cardiovascular event (> 90 days after therapy initiation), while earlier events were dominated by myocarditis and arrhythmias [24]. In a meta-analysis of 29,529 patients with a follow-up of 6.6 to 32.8 months, the incidence of cardiovascular adverse events under ICI ranges from 3.2 (95% CI 2.0–5.1) to 19.3 (6.7–54.1) per 1000 patients. The highest risk ratio for the ICI population compared to the control population were for dyslipidemia, followed by myocarditis, pericardial disease, heart failure, ischemic stroke and myocardial infarction [13]. 

However, asymptomatic CTRCD may be significantly more common, with reported rates ranging from 8.5% to 31.3% in ICI-treated patients across two studies [15, 17]. These findings raise concerns about delayed cardiac complications that may be underrecognized in routine clinical practice. The incidence of 2.63% HFpEF in our study group is similar to other studies in the literature [14, 15]. A meta-analysis on patients with non-small cell lung cancer treated with PD-1 or PDL-1 inhibitors showed a 2% incidence of cardiac dysfunction (95% CI, 1.0–5.7%) [14]. Data from JAVELIN trial, which compared axitinib plus avelumab vs. sunitinib in renal cell carcinoma, showed an incidence of heart failure of 1.6% in the avelumab group, compared to 0.7% in the sunitinib group [15]. 

The use of GLS as early indicator of CTRCD did not show promising results in the SUCCOUR study, where the difference in LVEF from baseline to 1 year being − 3.0% in the LVEF-guided arm versus − 2.7% in the GLS-guided arm [25]. Given the significant cardiovascular risks associated with ICI therapy, our findings highlight the importance of GLS in the diagnostic of CTRCD. Future research should focus on determining the prognostic significance of asymptomatic mild CTRCD and HFpEF and whether early intervention improves outcomes. Identifying predictive biomarkers and imaging indicators of ICI-related cardiovascular toxicity, evaluating the long-term cardiovascular effects of ICI therapy and developing effective cardioprotective strategies are urgent priorities; notably, our preclinical data indicate that TNFα blockade preserves left ventricular function without diminishing anti-tumor efficacy [19]. 

Although definitive prognostic implications of asymptomatic CTRDC are not yet established, several considerations support our interpretation. A ≥ 15% reduction in GLS is a well-validated early marker of subsequent LVEF decline in other cardiotoxic settings, including anthracyclines and anti-HER2 therapies. In our cohort, patients with eirAEs exhibited a nine-fold higher risk of developing HFpEF, suggesting that these early myocardial changes may help identify a vulnerable, inflammation-driven phenotype. Importantly, early detection provides an opportunity to adjust the ICI treatment regimen and implement preventive cardioprotective strategies, potentially mitigating progression to overt cardiac dysfunction.

Our findings underscore the potential clinical relevance of identifying early cardiac alterations, especially in patients who manifest extracardiac immune toxicities. Whether such early changes translate into progressive dysfunction or symptomatic heart failure beyond the first year remains unknown. Prospective multicenter registries with harmonized imaging and biomarker strategies, as well as longer follow-up, will be essential to address these gaps and to determine which patient subgroups benefit most from intensified cardiovascular surveillance. Ultimately, integrating immune-related toxicity profiles with longitudinal cardiac assessments may enable a more personalized approach to monitoring and risk stratification in patients receiving ICIs.

### Limitations

This study is a retrospective analysis of real-world patients from our EcoR database who received ICI therapy. Retrospective studies inherently carry the risk of selection bias and incomplete data collection, which may affect the generalizability of our findings.

Although 520 patients undergoing ICI therapy were recorded in the EcoR database, only 266 met the inclusion criteria due to the lack of a baseline cardio-oncology assessment prior to ICI initiation. The absence of baseline and longitudinal cardiac imaging in these patients would have limited our ability to reliably determine the true incidence of CTRCD. This exclusion may have led to a selection bias. Referral criteria have included perceived cardiovascular risk, availability of cardio-oncology services at the time of ICI initiation, or treating physician discretion.

Not all patients underwent follow-up examinations in our clinic (14%), leading to potential loss of data regarding long-term cardiovascular outcomes. Patients who discontinued follow-up or sought evaluation at external institutions were not systematically tracked, which may have affected the accuracy of reported incidence rates for CTRCD and myocarditis. Strain measurements were unavailable for 20 patients (7.5%), and NT-proBNP values were missing for 22 patients (8.2%) Strain measurements were conducted by one experienced and certified operator. Intra-observer and inter-observer variability of the echocardiography parameter was not systematically analyzed due to the retrospective design of the study.

This study was conducted in a single cardio-oncology center, which may limit the external validity of our results. The patient population may differ from those in other institutions, particularly regarding baseline cardiovascular risk factors, cancer types, and treatment regimens. A multicenter study would provide more generalizable findings. Additionally, as some studies suggest that ICI-related cardiovascular toxicity may manifest months or years after treatment, our one-year follow-up duration may not be sufficient to capture long-term cardiac dysfunction.

A non-ICI-treated control group was not included in our study, limiting our ability to differentiate the effects of ICI therapy from other confounding factors, such as cancer progression, prior chemotherapies, or radiation-induced cardiotoxicity. A matched control group of cancer patients not receiving ICIs would strengthen the causal inference between ICI therapy and CTRCD.

To address these limitations, larger prospective, multicenter studies with systematic cardiac biomarker assessments, extended follow-up periods, and control groups should be conducted. Additionally, machine learning models may help predict which patients are at highest risk for ICI-related cardiotoxicity, guiding personalized surveillance strategies.

## Conclusion

In this cohort of patients receiving immune checkpoint inhibitors, we observed that guideline-defined mild, asymptomatic CTRCD is substantially more frequent than previously appreciated, particularly in patients who develop extracardiac immune-related adverse events.

The high incidence of asymptomatic CTRCD highlights the risk of silent cardiovascular toxicity that can only be identified through routine echocardiographic surveillance—yet current guidelines recommend such imaging only for high-risk patients at baseline. Our findings therefore challenge these restrictive echocardiography recommendations for cancer patients receiving ICI therapy. In particular, patients with eirAEs may benefit from closer cardiac monitoring, as they appear to exhibit a higher susceptibility to CTRCD. Accordingly, we propose that future studies and guideline updates should focus on better defining such high-risk subgroups, in whom targeted echocardiographic surveillance could be justified to enable early detection and potential intervention, ultimately supporting a more individualized, risk-adapted surveillance strategy rather than a uniform approach. However, the prognostic implications of mild CTRCD in the ICI setting remain uncertain, and our findings should therefore be interpreted with caution. Nevertheless, the high incidence of early subclinical dysfunction highlights the potential value of structured cardiovascular monitoring, especially in patients with concomitant immune-related toxicities.

## Supplementary Information

Supplementary Material 1.

## Data Availability

No datasets were generated or analysed during the current study.

## Abbreviations

CTLA-4: Cytotoxic T-lymphocyte antigen 4
CTRCD: Cancer therapy related cardiac dysfunction
ESC: European Society of Cardiology
GLS: Global longitudinal strain
HFpEF: Heart failure with preserved ejection fraction
ICI: Immune checkpoint inhibitors
eirAEs: Extracardiac immune-related adverse events
LVEF: Levft ventricular ejection fraction
NT-proBNP: N-terminal pro–B-type natriuretic peptide
PD-1: Programmed cell death protein 1
PD-L1: Programmed death-ligand 1
RR: Relative risk
TTE: Transthoracic echocardiography
