# Supplementary material for: Cardiac dysfunction during immune checkpoint inhibitor therapy: association with extracardiac immune-related adverse events
Source: Cardiooncology. 2026 Jun 16;12:77. doi: 10.1186/s40959-026-00529-4 (PMC13270561; doi:10.1186/s40959-026-00529-4)
Supplement: Supplementary file 1 — Supplementary Material 1. [file 40959_2026_529_MOESM1_ESM.docx]

***Cardiac Dysfunction During Immune Checkpoint Inhibitor Therapy: Association With Extracardiac Immune-Related Adverse Events***

**Supplemental appendix**

Raluca I. Mincu, M.D., Ph.D.^a^, Lena Lampe ^a^, Lars Michel, M.D.^a^, Amir A. Mahabadi, M.D.^a^, Adelina V. Mark^a^, Lisa Zimmer, M.D.^b^, Elisabeth Livingstone M.D.^b^, Dirk Schadendorf, M.D.^b^, Alpaslan Tasdogan, M.D., Ph.D. ^b^, Tienush Rassaf, M.D.^a^, and Matthias Totzeck, M.D.^a^

a: Department of Cardiology and Vascular Medicine, West German Heart and Vascular Center Essen, University Hospital Essen, Essen, Germany;

b: Department of Dermatology, West German Skin Tumor Center Essen, University Hospital Essen, Essen, Germany.

**Corresponding Author:**

Associate Professor Dr. Raluca-Ileana Mincu, PhD

University Hospital Essen

West German Heart and Vascular Center Essen

Department of Cardiology and Vascular Medicine

Hufelandstrasse 55, 45147 Essen, Germany

Tel: +49 201 723 83720; Fax: +49 201 5401

Email: [Raluca-Ileana.Mincu@uk-essen.de](mailto:Raluca-Ileana.Mincu@uk-essen.de)

Supplementary Table 1. Definition of cardiovascular toxicities of cancer therapies according to the International Cardio-Oncology Society (IC-OS) consensus statement from the cardio-oncology guidelines. ^1, 2^

| **Cardiotoxicity type** | **Category** | **Severity** | **Diagnosis criteria** |
| --- | --- | --- | --- |
| **Cancer Therapy Related Cardiac Dysfunction (CTRCD)** | **Asymptomatic**  (with or without additional biomarkers, LVEF values are based on 2D echocardiography) | *Mild* | LVEF ≥ 50%  AND new relative decline in GLS by > 15% from baseline  AND/OR new rise in cardiac biomarkers (Troponin I/T > 99^th^ percentile, BNP ≥ 35 pg/ml, NT-proBNP ≥ 125 pg/ml) |
|  |  | *Moderate* | New LVEF reduction by ≥ 10 percentage points to an LVEF of 40-49%  OR  New LVEF reduction by < 10 percentage points to an LVEF of 40-49%  AND new relative decline in GLS by >15% from baseline  AND/OR new rise in cardiac biomarkers |
|  |  | *Severe* | New LVEF reduction to <40% |
|  | **Symptomatic**  Symptomatic CTRCD represents HF, which is a clinical syndrome consisting of cardinal symptoms (e.g. breathlessness, ankle swelling, and fatigue) that may be accompanied by signs (e.g. elevated jugular venous pressure, pulmonary crackles, and peripheral oedema) and has traditionally been divided into distinct phenotypes based on the measurement of LVEF: ≤ 40%= HFrEF; 41–49 % = HFmrEF; ≥ 50 % = HFpEF. | *Mild* | Mild HF symptoms, no intensification of therapy required |
|  |  | *Moderate* | Need for Outpatient intensification of diuretic and HF therapy |
|  |  | *Severe* | HF Hospitalisation |
|  |  | *Very severe* | Requiring inotropic support, mechanical circulatory support or consideration for transplantation |
| **Myocarditis** | **Pathohistological diagnosis**  Multifocal inflammatory cell infiltrates with overt cardiomyocyte loss by light microscopy of cardiac tissue samples.  **or**  **Clinical diagnosis**  A troponin elevation with 1 major criterion  or  a troponin elevation with 2 minor criteria after exclusion of acute coronary syndrome or acute infectious myocarditis based on clinical suspicion  **Major Criterion**  CMR diagnostic for acute myocarditis (modified Lake Louise criteria)  **Minor Criteria**   - Clinical syndrome - Ventricular arrhythmia and/or new conduction system disease - Decline in cardiac function - Other immune-related adverse events - Suggestive CMR | *Fulminant* | Hemodynamic instability, heart failure requiring non-invasive or invasive ventilation, complete or high-grade heart block, and/or significant ventricular arrhythmia |
|  |  | *Non-fulminant (clinically significant*) | Symptomatic but hemodynamically and electrically stable patients and incidental cases diagnosed at the the same time as other immuno-related adverse events. Patients may have reduced LVEF, but no features of severe disease.  Incidentally diagnosed myocarditis without any clinical signs or symptoms |
|  |  | *Steroid refractory* | Non-resolving or worsening myocarditis (clinical worsening or persistent troponin elevation after exclusion of other etiologies) despite high-dose methylprednisolone |
| **Vascular Toxicity** | **Asymptomatic** | *Atherosclerosis* | Coronary artery disease: New coronary artery stenosis >50% on coronary computed tomography (CT) angiogram or >70% on coronary angiogram, or newly abnormal electrocardiogram (ECG), nuclear or echo stress test Peripheral arterial disease: New ankle-brachial index (ABI) value ≤ 0.9 is considered abnormal, with 0.7-0.9 being mildly reduced, 0.4-0.69 moderately reduced, and < 0.4 severely reduced or Chance in ABI from baseline by -0.15 Carotid artery disease: New intima media thickness (IMT) > 0.9 mm or new plaque on carotid ultrasound, or Change in IMT > 0.04/year from baseline |
|  |  | *Thrombosis* | Venous thrombosis: New characteristic features on Duplex ultrasound, contrast CT, or venogram  Arterial thrombosis: New characteristic features on ultrasound or angiogram, or optical coherence tomography |
|  |  | *Abnormal* *vasoreactivity* | Peripheral: New flow-mediated dilation of the brachial artery (FMD) < 7.1% or reactive hyperemia index (RHI) < 2 on Endo-PAT, or Change in FMD or RHI by > 50% from baseline.  Coronary epicardial: New coronary vasoconstriction (reduction in coronary artery diameter) in response to acetylcholine infusion. Coronary microvascular: New < 50% increase in coronary blood flow in response to acethylcholine infusion, or a coronary flow reserve < 2 in response to adenosine. |
|  | **Symptomatic** | *Stroke* | 2018 AHA/ASA Guidelines for the Early Management of Patients with Ischemic Stroke  An Updated Definition of Stroke for the 21st Century Stroke |
|  |  | *Transient ischemic attack* |  |
|  |  | Myocardial infarction | 4^th^ Universal Definition of Myocardial infarction |
|  |  | Acute coronary syndromes | 2013 ACCF/AHA Guideline for the Management of ST-Elevation Myocardial Infarction82 2014 AHA/ACC Guideline for the Management of Patients with Non–ST-Elevation Acute Coronary Syndromes  2015 ESC Guidelines for the management of acute coronary syndromes in patients presenting without persistent ST-segment elevation  2017 ESC Guidelines for the management of acute myocardial infarction in patients presenting with ST-segment elevation8 |
|  |  | *Chronic coronary syndromes* | 2019 ESC Guidelines for the diagnosis and management of chronic coronary syndromes: The Task Force for the diagnosis and management of chronic coronary syndromes of the European Society of Cardiology (ESC) |
|  |  | *Peripheral arterial disease* | 2017 ESC Guidelines on the Diagnosis and Treatment of Peripheral Arterial Diseases, in collaboration with the European Society for Vascular Surgery (ESVS |
|  |  | *Vasospastic angina* | 2019 ESC Guidelines for the diagnosis and management of chronic coronary syndromes: The Task Force for the diagnosis and management of chronic coronary syndromes of the European Society of Cardiology (ESC)  International standardization of diagnostic criteria for vasospastic angina |
|  |  | *Microvascular angina* | 2019 ESC Guidelines for the diagnosis and management of chronic coronary syndromes: The Task Force for the diagnosis and management of chronic coronary syndromes of the European Society of Cardiology (ESC)  International standardization of diagnostic criteria for microvascular angina |
|  |  | *Raynaud’s phenomenon* | Meeting the diagnostic criteria of an international consensus panel of recurrent episodes bilateral blanching or tricolor change of the fingers. |
|  |  | *Hypertensive emergency response* | Very high BP elevations associated with acute hypertension-mediated organ damage (heart, retina, brain, kidneys, and large arteries), therefore, requiring immediate BP reduction to limit extension or promote regression of target organ damage |
| **QT Prolongation and arrhythmias** | **QTc prolongation** | *QTcF < 480ms* | Acceptable: continue current treatment |
|  |  | *QTcF 480-500ms* | Prolonging: proceed with caution; minimize other QT prolonging medications, replete electrolytes |
|  |  | *QTcF > 500ms* | Prolonged: stop treatment and evaluate. May require dose reduction or alternative therapy |
|  | **Arrhythmias** | *Ventricular arrhythmia* | 2015 ESC Guidelines for the management of patients with ventricular arrhythmias and the prevention of sudden cardiac death  2017 AHA/ACC/HRS Guideline for Management of Patients With Ventricular Arrhythmias and the Prevention of Sudden Cardiac Death |
|  |  | *Ventricular tachycardia (VT), including polymorphic VT (torsades de pointes)* |  |
|  |  | *Ventricular fibrillation* |  |
|  |  | *Atrial fibrillation* | 2020 ESC Guidelines for Management of Atrial Fibrillation  2014 AHA/ACC/HRS Guideline for the Management of Patients With Atrial Fibrillation |
|  |  | *Atrial flutter* |  |
|  |  | *Atrial tachycardia* | 2019 ESC Guidelines on Supraventricular Tachycardia  2015 ACC/AHA/HRS Guideline for the Management of Adult Patients With Supraventricular Tachycardia: A Report of the American College of Cardiology/American Heart Association Task Force on Clinical Practice Guidelines and the Heart Rhythm Society |
|  |  | *Supraventricular tachycardia* |  |
|  |  | *Sinus tachycardia* |  |
|  |  | *Sinus bradycardia* | 2018 ACC/AHA/HRS Guideline on the evaluation and management of patients with bradycardia and cardiac conduction delay |
|  |  | *Sick sinus syndrome* |  |
|  |  | *Atrioventricular block first, second and third degree* |  |
|  |  | *Conduction disorder (disease)* |  |

ACC = American College of Cardiology. AHA = American Heart Association. ASCVD = atherosclerotic cardiovascular disease. ASE = American Society of Echocardiography. BP = blood pressure. CMR = cardiac magnetic resonace. CTRCD = Cancer-therapeutics Related Cardiac Dysfunction. DBP = diastolic blood pressure. GLS = Global Longitudinal Strain. HF = Heart Failure. LVEF = left ventricular ejection fraction. QTcF = QT interval corrected by the Fridericia formula. SBP = systolic blood pressure.

Supplementary Table 2 Extracardiac immune related adverse events (eirAE) in the study population.

The table shows the distribution of extracardiac immune related adverse events in the whole population. Values are expressed in number of patients (percent of total patient population) n (%).

| eirAEs | N (%)  n = 266 |
| --- | --- |
| Any eirAEs n (%) | 104 (39.0) |
| Hepatitis n (%) | 41 (15.4) |
| Colitis n (%) | 36 (13.5) |
| Thyroiditis n (%) | 19 (7.1) |
| Hypophysitis n (%) | 12 (4.5) |
| Pneumonitis n (%) | 11 (4.1) |
| Myositis n (%) | 4 (1.5) |
| Gastroenteritis n (%) | 2 (0.7) |
| Nephritis n (%) | 2 (0.7) |
| Uveitis n (%) | 1 (0.3) |
| Leukopenia n (%) | 1 (0.3) |
| Pankreatitis n (%) | 1 (0.3) |

Supplementary Table 3. LVEF and Strain at three different timepoints in each of the two study groups.

The table shows the dynamics of LVEF, GLS, GRS and GCS between the four evaluation timepoits at baseline, 6 week (6 w), 6 months (6 m) and 12 months (12 m) follow-up examinations in each of the two study groups. Data represent mean ± SD.

eirAEs = immune related adverse events; GCS = global circumferential strain; GLS = global longitudinal strain; GRS = global radial strain

|  | Baseline  n = 266 | 6 weeks | 6 months | 12 months | p-value  Base-line vs. 6 w | p-value  Base-line vs. 6 m | p-value  Base-line vs. 12 m |
| --- | --- | --- | --- | --- | --- | --- | --- |
| 2D LVEF (%) | | | | | | | |
| Group 1  eirAEs | 59.55 ± 7.43 | 58.91 ± 5.79 | 60.19 ± 6.77 | 59.18 ± 6.35 | 0.460 | 0.848 | 0.166 |
| Group 2  no eirAEs | 59.89 ± 6.57 | 59.30 ± 6.65 | 59.26 ± 5.09 | 59.65 ± 5.15 | 0.270 | 0.248 | 0.323 |
| 3D LVEF (%) | | | | | | | |
| Group 1 eirAEs | 60.37 ± 5.17 | 62.28 ± 3.89 | 59.17 ± 4.78 | 61.4 ± 4.33 | 0.100 | 0.608 | 0.087 |
| Group 2  no eirAEs | 61.21 ± 6.7 | 60.31 ± 6.34 | 59.52 ± 4.21 | 58.68 ± 5.63 | 0.312 | 0.911 | 0.551 |
| GLS (%) | | | | | | | |
| Group 1 irAEs | -20.47 ± 2.47 | -18.60 ± 3.77 | -18.88 ± 2.29 | -18.57 ± 5.95 | <0,001 | <0,001 | 0.009 |
| Group 2  no eirAEs | -20.24 ± 2.84 | -20.05 ± 2.24 | -19.76 ± 2.10 | -19.47 ± 1.94 | 0.514 | 0.365 | 0,060 |
| GRS (%) | | | | | | | |
| Group 1 eirAEs | 59.24 ± 5.30 | 58.00 ± 5.35 | 58.36 ± 6.57 | 57.71 ± 4.98 | 0.113 | 0.874 | 0.131 |
| Group 2  no eirAEs | 58.29 ± 7.56 | 56.97 ± 7.61 | 59.17 ± 6.01 | 58.07 ± 4.11 | 0.112 | 0.412 | 0.264 |
| GCS (%) | | | | | | | |
| Group 1 eirAEs | -27.87 ± 6.48 | -27.56 ± 5.66 | -27.40 ± 6.33 | -25.68 ± 5.03 | 0.822 | 0.716 | 0.136 |
| Group 2  no eirAEs | -29.26 ± 7.18 | -29.02 ± 7 | -28.84 ± 6.39 | -28.69 ± 6.2 | 0.865 | 0.304 | 0.787 |
| NT-proBNP (pg/ml) | | | | | | | |
| Group 1 eirAEs | 163 (67.6 – 659) | 470 (182 – 2624) | 153 (54 – 404) | 206.5 (89.75 – 619.5) | 0.295 | 0.344 | 0.028 |
| Group 2  no eirAEs | 306 (109.75 – 909.75) | 369 (109.75 – 909.75) | 206 (149 – 1207) | 175 (119 – 460) | 0.292 | 0.225 | 0.776 |

Supplementary Figure 1.

Kaplan Meyer survival curves for the ICI therapy-naïve patients (266 patients) in blue vs. ICI non-threapy-naïve patients (254 patients) in green. Survival data for the 254 excluded patients under ICI. The survival curve of ICI therapy–naive patients differed significantly from that of non–therapy-naive patients. After one year, 86.5% of therapy-naive patients were still alive, wheareas only 77.8% of non–therapy-naive patients were alive (p = 0.002).

ICI = immune checkpoint inhibitors;


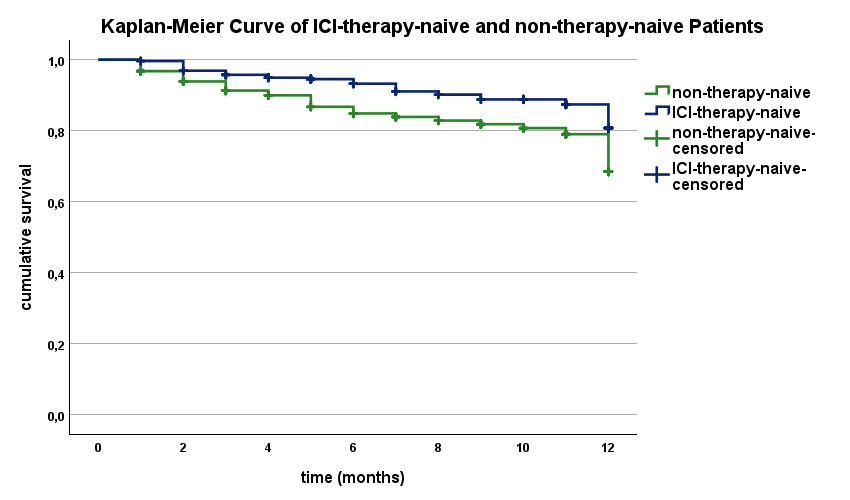


**p=0.002**

**Mean survival**

**ICI-therapy-naive patients**

**11.21 ± 0.15 (MV ± SEM)**

**Non-therapy-naive patients**

**10.52 ± 0.21(MV ± SEM)**

References

1. Herrmann J, Lenihan D, Armenian S, Barac A, Blaes A, Cardinale D, Carver J, Dent S, Ky B, Lyon AR, López-Fernández T, Fradley MG, Ganatra S, Curigliano G, Mitchell JD, Minotti G, Lang NN, Liu JE, Neilan TG, Nohria A, O'Quinn R, Pusic I, Porter C, Reynolds KL, Ruddy KJ, Thavendiranathan P and Valent P. Defining cardiovascular toxicities of cancer therapies: an International Cardio-Oncology Society (IC-OS) consensus statement. *Eur Heart J*. 2022;43:280-299.

2. Lyon AR, López-Fernández T, Couch LS, Asteggiano R, Aznar MC, Bergler-Klein J, Boriani G, Cardinale D, Cordoba R, Cosyns B, Cutter DJ, de Azambuja E, de Boer RA, Dent SF, Farmakis D, Gevaert SA, Gorog DA, Herrmann J, Lenihan D, Moslehi J, Moura B, Salinger SS, Stephens R, Suter TM, Szmit S, Tamargo J, Thavendiranathan P, Tocchetti CG, van der Meer P and van der Pal HJH. 2022 ESC Guidelines on cardio-oncology developed in collaboration with the European Hematology Association (EHA), the European Society for Therapeutic Radiology and Oncology (ESTRO) and the International Cardio-Oncology Society (IC-OS). *Eur Heart J*. 2022;43:4229-4361.
